# Effect of Submaximal Doses of Semaglutide in Patients with Obesity on Metabolic Profile and Serum Levels of Adipocytokines

**DOI:** 10.3390/ph18091364

**Published:** 2025-09-12

**Authors:** Martin Jozef Péč, Jakub Jurica, Monika Péčová, Norbert Nagy, Boris Focko, Zuzana Miertová, Nikola Ferencová, Ivana Ságová, Ingrid Tonhajzerová, Tomáš Bolek, Peter Galajda, Marián Mokáň, Matej Samoš

**Affiliations:** 1 Department of Internal Medicine I., Jessenius Faculty of Medicine in Martin, Comenius University in Bratislava, 03659 Martin, Slovakia; pec5@uniba.sk (M.J.P.); kubo.jurica@gmail.com (J.J.); norbi.nagy.nn@gmail.com (N.N.); borisfo@gmail.com (B.F.); moravcikova25@uniba.sk (Z.M.); ivana.sagova@uniba.sk (I.S.); peter.galajda@uniba.sk (P.G.); mokanmarian@gmail.com (M.M.); 2 Department of Hematology and Transfusiology, Jessenius Faculty of Medicine in Martin, Comenius University in Bratislava, 03659 Martin, Slovakia; kucerikovam@gmail.com; 3 Oncology Centre, Teaching Hospital Martin, 03659 Martin, Slovakia; 4 Department of Physiology, Jessenius Faculty of Medicine in Martin, Comenius University in Bratislava, 03601 Martin, Slovakia; nikola.ferencova@uniba.sk (N.F.); ingrid.tonhajzerova@uniba.sk (I.T.); 5 Department of Endocrinology, National Institute of Endocrinology and Diabetology, 03491 Ľubochňa, Slovakia; 6 Department of Acute and Interventional Cardiology, Mid-Slovakian Institute of Heart and Vessel Diseases (SÚSCCH), 97401 Banská Bystrica, Slovakia

**Keywords:** obesity, semaglutide, adipocytokines, glucagon-like peptide-1 receptor agonists, metabolic profile

## Abstract

**Background:** Obesity is closely linked to metabolic dysfunction and systemic low-grade inflammation. Glucagon-like peptide-1 receptor agonists (GLP-1RA) are increasingly utilized for obesity treatment due to their significant metabolic benefits, including weight loss and improved glycemic control. The aim of the study was to evaluate the effect of submaximal doses of long-lasting GLP-1RA semaglutide on selected biomarkers of obesity-related inflammation, adipocytokines levels and metabolism in a real-world population of obese patients. **Methods:** We performed a prospective, observational study involving 32 adult patients (11 men, 21 women; mean age 49 ± 12 years; BMI 40.5 ± 7.3 kg/m^2^) treated with submaximal doses of semaglutide over 12 weeks, together with hypocaloric diet and increased physical activity based. We analyzed selected biomarkers including insulin, leptin, ferritin, resistin, interleukin-6 (IL-6), tumor necrosis factor-alpha (TNF-α) and plasminogen activator inhibitor-1 (PAI-1) before and after three months of treatment. **Results:** We observed significant reductions in weight, BMI, waist circumference, insulin and leptin levels (all *p* < 0.001). On the other hand, no significant changes were recorded in ferritin (*p* = 0.806), IL-6 (*p* = 0.607), TNF-α (*p* = 0.633), resistin (*p* = 0.250) or PAI-1 (*p* = 0.134) levels. Correlation analyses revealed the correlation between IL-6 and adiposity indices (BMI, waist circumference) both before and after treatment. Ferritin and PAI-1 levels positively correlated with waist circumference, while resistin showed a negative correlation with central obesity. **Conclusions:** Submaximal-dose GLP-1 RA therapy was associated with significant improvements in metabolic parameters and adipokine regulation, but did not affect systemic inflammatory markers within 12 weeks. Future studies with larger cohorts and longer follow-ups are needed to clarify the associations.

## 1. Introduction

Obesity is one of the most significant global health challenges of our time, with widespread consequences for health, society and the economy [1]. It is closely connected to metabolic dysregulation and chronic subclinical inflammation [2,3]. These connections are increasing the risk of diabetes, cardiovascular diseases and other associated diseases. Although decreased energy intake, increased physical activity and cognitive-behavior therapy (so-called life-style change modifications) remain the cornerstones of obesity therapy, one must say that long-term effects of these therapeutical approaches still do not obtain good results. As demonstrated in the meta-analysis performed by Nordmo et al. [4], multiple studies demonstrate the trend of weight regain towards pretreatment baseline if only life-style modification is used to treat obesity. On the other hand, glucagon-like peptide-1 receptor agonists (GLP-1RAs) represent a newer group of medications used to treat obesity, showing significant potential in supporting weight loss for individuals struggling with this condition [3]. These drugs work by regulating appetite, aiding in weight reduction and enhancing blood sugar control [5]. Looking at the role of GLP-1RAs in obesity management, first using short-acting agents, then replacing them with long-acting ones, demonstrated truly significant and long-term effects on weight reduction when administered in anti-obesity (maximal) doses in adults, as well in children and adolescents [5,6,7,8,9,10,11]. The results of the marketing studies with GLP-1RAs [5,6,7,8,9,10,11] established GLP-1RAs as the most frequently used drugs for the treatment of obesity, and established anti-obesity pharmacotherapy as another cornerstone in the management of obesity, representing a true revolution in the treatment of obesity. Beyond their metabolic advantages, recent studies indicate that GLP-1RAs might also influence systemic inflammation and alter the levels of important biomarkers linked to obesity [12]. Understanding the impact of GLP-1RAs on these biomarkers may shed light on the mechanisms underlying their therapeutic effects and expand their utility in managing obesity and associated metabolic disorders. At the same time, the research tries to identify molecular biomarkers that can serve as potential markers of successful treatment. Biomarkers, such as ferritin, leptin, resistin, interleukin-6 (IL-6), insulin, tumor necrosis factor-alpha and plasminogen activator inhibitor-1 (PAI-1), play crucial roles in the pathophysiology of obesity [13]. These are markers of inflammation and metabolic health and potential therapeutic targets for intervention. Ferritin is a protein of the acute phase that is elevated during inflammation. Chronic inflammation associated with obesity stimulates the production of ferritin [13]. Furthermore, in obese patients, leptin levels are elevated, but, paradoxically, this elevation does not lead to a decrease in appetite or body weight regulation due to leptin resistance. Increased leptin levels are connected to decreased insulin sensitivity. This can worsen metabolic capability and lead to the development of type 2 diabetes, subsequently further stimulating chronic inflammation [14]. Obese patients have elevated levels of resistin. Resistin plays a role in the development of insulin resistance by interfering with the glucose-regulating activity of insulin [15]. Elevated resistin levels are associated with increased risk of atherosclerosis and hypertension [16]. In obese patients, chronic low-grade inflammation is present, and is closely associated with visceral adipose tissue. In patients with obesity, IL-6 levels correlate with inflammation [17]. IL-6 produced by skeletal muscles reduces glucose production [18], improves insulin resistance and improves beta cell function and glucose homeostasis [19]. Hyperinsulinemia is often observed in obese patients as a compensatory response to insulin resistance. Chronically elevated insulin levels contribute to the maintenance of obesity by promoting lipogenesis in adipose tissue and inhibiting lipolysis [20]. Tumor necrosis factor alpha (TNF-α) is elevated in obese patients, primarily due to its production by macrophages in adipose tissue, which contributes to chronic low-grade inflammation [21]. TNF-α plays a central role in the pathogenesis of obesity by disrupting insulin signaling, promoting insulin resistance and metabolic dysfunction [2,3]. Plasminogen activator inhibitor-1 (PAI-1) is elevated in obese patients [22]. It appears that hyperinsulinemia promotes the expression of PAI-1, the regulator of fibrinolysis, which contributes to the pathogenesis of obesity [23]. Summarizing these observations, one could suggest that successful GLP1-RA therapy and successful weight reduction would lead to a significant change in plasma levels of the above-mentioned markers. As there is no study directly examining the effect of submaximally dosed semaglutide therapy on plasma levels of selected inflammatory, metabolic biomarkers and adipocytokines associated with obesity, the aim of the study was to assess the effect of 12 weeks (3 months) of treatment with submaximal doses of GLP-1RA in non-diabetic patients with obesity. Furthermore, based on the collected data, we aimed to identify relationships between treatment efficacy and biological indicators, which could create strategies for the more personalized therapy of obesity.

## 2. Results

### 2.1. Patients and Baseline Metabolic Profile

During the study period, we prospectively included 32 patients (11 men, 21 women) with a mean age of 49 ± 12 years. Dyslipidemia was observed in 84.4% of patients. Other comorbidities included hypertension (53.1%), coronary artery disease (21.9%) and chronic kidney disease (9.4%). Baseline parameters are summarized in Table 1, demonstrating a mean serum creatinine of 71.9 ± 12.3 µmol/L and an estimated glomerular filtration rate (GFR) of 94.6 ± 14.2 mL/min/1.73 m^2^. Lipid profile consisted of elevated total cholesterol (5.6 ± 1.1 mmol/L), LDL cholesterol (3.5 ± 1.1 mmol/L) and triglycerides (2.0 ± 1.0 mmol/L), with an HDL cholesterol level of 1.2 ± 0.3 mmol/L.

Before treatment, we observed a positive correlation between IL-6 and BMI (r = 0.465, *p* = 0.008), IL-6 and waist circumference (r = 0.492, *p* = 0.005) and IL-6 and waist-to-height ratio (r = 0.592, *p* < 0.001). Also, we found a positive correlation between weight and serum creatinine (r = 0.418, *p* = 0.014) and waist circumference and serum creatinine (r = 0.385, *p* = 0.024) before treatment. Results showed a negative correlation between total cholesterol and BMI (r =−0.485, *p* = 0.004), total cholesterol and waist circumference (r =−0.369, *p* = 0.032) and total cholesterol and waist-to-height ratio (r =−0.352, *p* = 0.041).

### 2.2. Effect of Submaximal Semaglutide on Metabolic Profile, Adipocytokines and Inflammation

**Figure 1:**
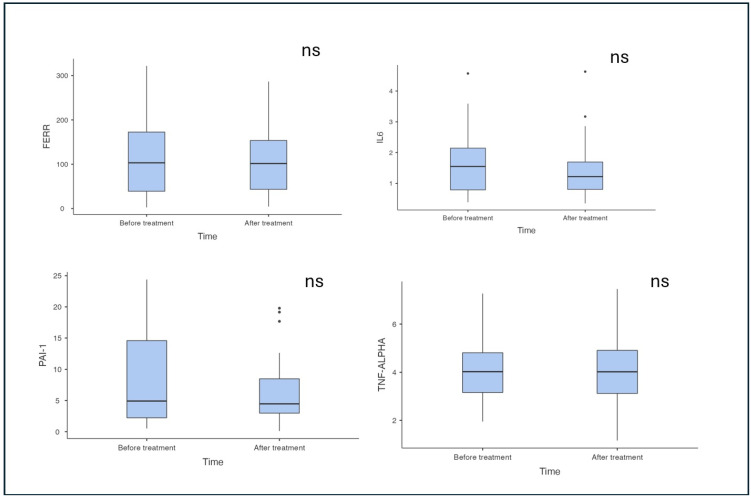
The effect of submaximally dosed semaglutide therapy on inflammatory markers in patients with obesity. IL—interleukin; FERR—ferritin; PAI—plasminogen activator inhibitor; TNF—tumor necrosis factor; *—*p* < 0.05; **—*p* < 0.01; ns—not significant.

**Figure 2:**
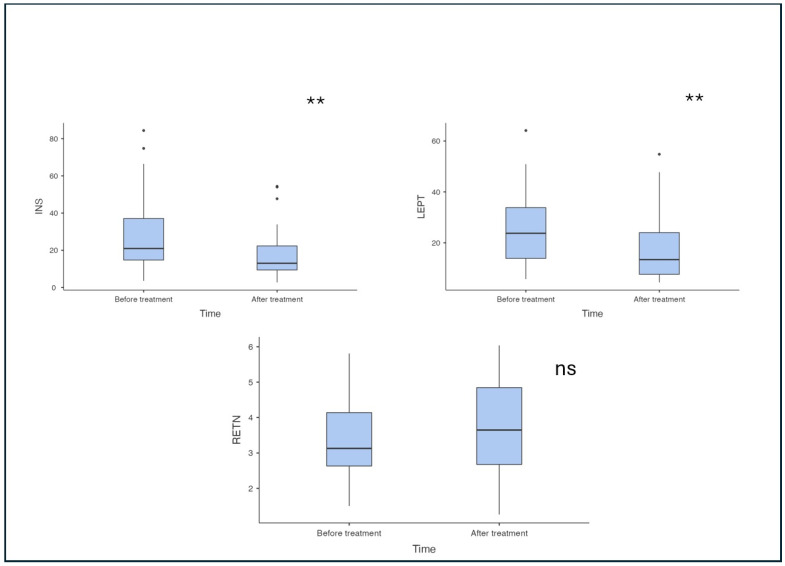
The effect of submaximally dosed semaglutide therapy on insulin levels and adipocytokines in patients with obesity. INS—insulin; LEPT—leptin; RETN—resistin; *—*p* < 0.05; **—*p* < 0.01; ns—not significant.

Following the intervention (Figure 1 and Figure 2), we observed significant metabolic changes. Insulin levels decreased markedly from 21.6 (15.1–42.3) uIU/mL to 13 (9.39–22.3) uIU/mL (*p* < 0.001). Similarly, a significant reduction in leptin levels was described from 24.6 (15.3–35.7) ng/mL to 14.1 (7.71–27.1) ng/mL. Also, we noticed the significant change in anthropometric parameters with improvement—weight, BMI and waist circumference decrease (*p* < 0.001). On the other hand, inflammatory markers including ferritin, IL-6, TNF-alpha and PAI-1 did not exhibit significant change after treatment with respective *p*-values of 0.806, 0.607, 0.633 and 0.134 (Table 2). Also, the level of resistin remained without a significant change (*p* = 0.250).

After treatment, we found a positive correlation between weight and IL-6 (r = 0.416, *p* = 0.02), BMI and IL-6 (r = 0.440, *p* = 0.013), waist circumference and IL-6 (r = 0.449, *p* = 0.011) and waist-to-height ratio and IL-6 (r = 0.408, *p* = 0.023). We observed a positive correlation between BMI and leptin (r = 0.363, *p* = 0.041) and between ferritin levels and waist circumference (r = 0.376, *p* = 0.037). Our study found that waist circumference and PAI-1 positively correlated (r = 0.467, *p* = 0.008). On the other hand, we observed a negative correlation between waist circumference and resistin (r = −0.372, *p* = 0.039) and between waist-to-height ratio and resistin (r = −0.381, *p* = 0.035).

## 3. Discussion

Our study demonstrated that 12 weeks of GLP-1RA semaglutide administrated at submaximal doses demonstrated a broad range of metabolic benefits, extending findings from landmark trials and the recent literature. These are new data which were not published in any of the previously published studies. In our study, insulin and leptin levels showed significant improvements in patients with obesity treated with submaximal dosages of GLP-1RA. On the other hand, inflammatory markers such as ferritin, IL-6, TNF-α, resistin and PAI-1 remained constant during the study period. Our results expand the body of evidence regarding the effect of submaximal doses of GLP-1RA on the selected metabolic and inflammatory markers. We demonstrated that submaximal doses of semaglutide significantly reduce weight in patients with obesity, which corresponds to large-scale clinical trials [10,24]. Notably, the observed significant reduction in insulin levels suggests an improvement in insulin sensitivity. GLP-1RAs stimulate glucose-dependent insulin secretion and may alleviate hyperinsulinemia by improving insulin resistance [25]. This positive effect of GLP-1RA therapy on insulin secretion was previously demonstrated for liraglutide in a prospective study enrolling patients with type 2 diabetes and obesity [26], in which 14 weeks of liraglutide therapy led to the improvement of glucose control and insulin sensitivity and resistance parameters. In addition, significantly higher levels of adipocytokines visfatin and resistin were also observed after the therapy, and baseline visfatin levels negatively correlated with basal fasting plasma glucose. In another study, using an animal model (male mice fed with a high-fat diet), liraglutide therapy decreased serum levels and the transcript levels of leptin, as well as leptin-signaling inhibitory regulators [27]. The positive effect on serum insulin levels during the standard oral glucose tolerance test was previously demonstrated also for exenatide [28].

Compensatory response to insulin resistance is chronic hyperinsulinemia, which is primarily driven by adiposity and chronic systemic inflammation [29,30,31]. The restoration of metabolic balance reflected in the decrease in insulin concentrations after GLP-1RA administration is most likely due to the medication’s effects on appetite regulation, delayed stomach emptying and increased glucose-dependent insulin secretion [32,33]. Since insulin resistance and hyperinsulinemia are major contributors to type 2 diabetes and its consequences, the improvement in insulin dynamics looks clinically significant.

Furthermore, our results showed a significant reduction in leptin levels after GLP-1RA treatment. Leptin, an adipokine primarily secreted by adipose tissue, plays a crucial role in energy homeostasis by altering hypothalamic appetite regulation and satiety [14]. In patients with obesity, leptin levels are elevated, leading to leptin resistance, and the brain is not able to respond to satiety signals. Leptin sensitivity may be partially restored by weight loss brought on by GLP-1RAs, improving appetite control and metabolic balance according to the reported drop in leptin levels [32]. We confirmed that weight loss caused by submaximal doses of GLP-1RA (together with low-calorie diet and recommended standardized physical activity) is associated with a decrease in leptin levels. This data adds to the current literature that significant weight loss is connected to leptin level reduction [33]. The recent meta-analysis of randomized controlled trials similarly found that GLP-1RA significantly decreased leptin levels (weighted mean difference: −4.85 ng/mL, 95% CI −9.32, −0.38, *p* = 0.03) [34]. A recent study suggested that GLP-1 may modulate leptin-signaling pathways to inhibit appetite [35]. Reduced leptin concentrations are associated with better metabolic health. Our results support the idea that GLP-1RA may normalize the adipokine profile in correlation with weight loss.

Despite the well-known connection between obesity and chronic subclinical inflammation, we did not observe a significant reduction in the studied inflammatory markers (ferritin, IL-6, TNF- α resistin, PAI-1) following the treatment with submaximal GLP-1 RA. These data are in contrast with previous studies, which showed the anti-inflammatory effect of GLP-1 RA [36]. Diabetes pathogenesis can be linked to inflammation through IL-6 elevation, which also contributes to impaired insulin signaling and hepatic glucose output [25]. Experimental models have shown that GLP-1 RA decreases IL-6 and TNF-α production in adipose and macrophages [37]. On the other hand, short-term studies showed no effect of GLP-1 RA on the IL-6 and TNF-α levels (14-week liraglutide treatment in type 2 diabetes patients) [32]. This effect can be potentially mitigated through the reduction of adiposity and modulation of immune cell activity [38]. To explain these findings, it is necessary to add that we observed patients just for a 12-week period, which may be insufficient to observe a significant reduction in systemic inflammatory biomarkers. We observed a positive correlation between IL-6 and anthropometric parameters both before and after GLP-1 RA treatment. These findings connect IL-6 production and adipose tissue, as the IL-6 level rises in obesity and is closely connected to insulin resistance. Park et al. demonstrated that IL-6 correlates with BMI and waist circumference [39]. In our study, levels of IL-6 correlate before and also after treatment, which suggests that even patients who lost weight but remained heavier continued to have higher IL-6. The fact that IL-6 did not significantly decrease in our group despite the weight loss but still correlated with anthropometric parameters suggests that significant weight loss may not be enough to decrease adipose tissue inflammation.

In our study, we observed a positive correlation between ferritin levels and waist circumference. Ferritin is an acute-phase reactant often elevated in obesity and metabolic syndrome. A study of Finnish adults showed a positive correlation between waist circumference and serum ferritin levels in both genders [40]. The same authors reported that increases in serum ferritin levels over time are associated with the development of metabolic syndrome. These findings suggest that patients with greater visceral fat measured with waist circumference have higher inflammation levels, and that puts them at risk for cardiovascular and metabolic complications. Our results are consistent with the literature that elevated serum ferritin is associated with abdominal obesity. Epidemiological data also showed that elevated ferritin is linked to metabolic syndrome and insulin resistance [36]. This finding indicates that patients with greater waist circumference have greater inflammation or hepatic iron store dysregulation [41].

Additionally, we found negative correlations between resistin and obesity anthropometric parameters. This is a contradictory result compared to previous studies, which mentioned resistin as a link between adiposity and insulin resistance. In humans, resistin is produced by macrophages in adipose tissue. Studies showed that resistin is not necessarily related to the markers of adiposity—BMI, waist-to-hip ratio, fat mass or insulin resistance [42]. On the other hand, a meta-analysis of randomized controlled trials, including more than 1000 patients, investigated the effect of GLP-1 RA and showed a significant decrease in resistin levels (weighted mean difference: −1.40 ng/mL, 95% CI −2.78, −0.01, *p* = 0.05) [34]. Interestingly, it was observed that resistin correlates with BMI and waist circumference in non-obese patients, but this correlation disappears in overweight/obese patients [43]. Our results suggest that extreme central obesity patients have a resistin plateau, or even decrease, relative to other inflammatory signals. One argument is that other adipokines (such as IL-6, TNF-α and leptin) predominate in the inflammatory profile in higher obesity states, and resistin, which is more closely linked to immune cell activation, may not scale up further with additional adiposity.

PAI-1 was, in our analysis, positively correlated with waist circumference and linked central obesity to a prothrombotic state. PAI-1 is the main inhibitor of fibrinolysis, and higher levels impair the breakdown of clots and are linked to prothrombotic risk. Obesity raises the chance of developing thrombotic vascular disorders. In both people and animals, the function of fat storage and its impact on PAI-1 levels were examined in a study by Shimomura [44]. In human participants, they found a strong correlation between plasma PAI-1 and visceral fat area, but not with subcutaneous fat area. In obese rats, PAI-1 mRNA was found in both types of fat tissue, but it only rose in visceral fat as obesity progressed. According to these findings, visceral fat’s increased PAI-1 gene expression may raise plasma levels and contribute to the onset of vascular disease in visceral obesity [45,46]. In our study, a bigger waist circumference was associated with higher PAI-1, which means a tendency towards thrombosis and atherosclerosis. This has significant implications. Patients with abdominal obesity have a higher risk of cardiovascular events not only because of associated hypertension, dyslipidemia and hyperglycemia but also because of the pro-coagulant effect of high PAI-1. We found that GLP-1RA treatment did not significantly reduce PAI-1, which suggests the prothrombotic conditions connected to visceral fat may persist even after weight loss. The PAI-1 levels increasing on the level of metabolic syndrome-associated hypercoagulability link central obesity to thrombotic events (like myocardial infarction). Compared to lean patients, patients with obesity have a 1.5–2.5-fold higher risk of arterial thrombosis [47]. Conversely, in patients with class III obesity who underwent Roux-en-Y gastric bypass, a considerable decrease in PAI-1 levels was observed [41]. This reduction was correlated with changes in leptin levels and BMI [41]. Mashayekhi et al. conducted a randomized controlled trial and found that, after 14 weeks of treatment with liraglutide, PAI-1 levels significantly declined [48].

To observe a significant decrease in IL-6, TNF-α or resistin, a longer duration of treatment or more weight loss could be necessary. However, GLP-1RAs may still have tissue-level anti-inflammatory effects (such as lowering adipose tissue inflammation or macrophage activation) that are not entirely reflected by circulating levels, even in the absence of significant acute changes in these markers [49,50,51].

Finally, it is questionable whether the observed changes were achieved due to GLP-1RA administration or if at least some of them could be caused by a reaction to non-pharmacological life change (low-calorie diet and increased physical activity) modification which was recommended to the patients. If one looks at the effects of a low-/very-low-calorie diet on insulin sensitivity and inflammatory markers, a previous study showed that very-low-calorie diet in women with obesity and polycystic ovary syndrome reduced body weight, improved plasma fasting glucose and insulin sensitivity and decreased plasma levels of PAI-1 [52]. In other observations, a very-low-calorie diet induced weight loss (by 5.8 +/− 0.8 kg) and decreased plasma PAI-1 concentration [53]. Another study showed that a very-low-calorie diet significantly changed leptin and adiponectin levels, while TNFα, IL-6 and PAI-1 levels did not differ significantly at nine months [54]. In this study, a commercially available very-low-calorie diet led to more potent changes compared to a low-carbohydrate/high-protein diet. In contrast, a study performed by Siklova-Vitkova et al. [55] failed to demonstrate significant changes in hyperinsulinemia, TNFα and PAI-1 levels with a weight-reducing hypocaloric diet in women with obesity. These observations suggest that a very-low-calorie diet would probably be needed to achieve changes in metabolic/inflammatory markers, and that the data about these changes are quite inconsistent. In our patients, only a low-calorie diet was recommended, and there are no data supporting the effects of low-calorie diet on studied metabolic/inflammatory markers, as all the available studies used a very-low-calorie one. Furthermore, although low physical activity is associated with insulin resistance and increased inflammation [56], there is no interventional study showing that increased physical activity really affects insulin resistance, adipokine or inflammatory levels in an adult obese population (this was demonstrated only in normal body weight male adolescents) [57]. To confirm/quantify the effects of non-pharmacological interventions compared to GLP-1RA therapy on the metabolic/inflammatory profile and weight reduction, a study directly comparing these interventions needs to be performed (see Section 3.1).

### 3.1. Strengths and Limitations

Looking at the strengths of our study, we would like to point out the fact that this is the first study directly examining the effect of submaximal doses of semaglutide on biomarkers of obesity-related inflammation, adipocytokines levels and metabolism in individuals with obesity. In our study, a relatively short-term duration of submaximally dosed semaglutide led to significant changes in several of the examined adipocytokines, suggesting that early effects of the therapy on metabolic changes can be achieved by submaximal doses of semaglutide. In addition, the therapy was clinically effective, with weight reduction corresponding with reduction achieved in marketing clinical studies (which up-titrated the therapy to maximal doses) [6,7,8,9,10,11,24]. We would like to point out the fact that maximally dosed semaglutide therapy is associated with increased risk of side effects, leading to premature therapy discontinuation. For example, in the original marketing study with semaglutide [10], 4.5% of participants in the semaglutide-treated cohort discontinued the treatment due to gastrointestinal side effects (intolerance). In light of this, submaximally dosed therapy can achieve better treatment tolerance and adherence, with probably the same metabolic effect. Our results advocate for future studies regarding the use of submaximal doses of semaglutide for the treatment of obesity, especially in those patients who do not tolerate maximal ones.

Looking at the possible limitations of our analysis, firstly, the limitation is the small sample size and single-center design, which limits the generalizability and statistical power of this study. Next, as mentioned, three months of therapy may be inadequate to assess metabolic and inflammatory changes, and longer duration of therapy might be needed for the observation of changes in these parameters. Finally, we did not include a control group. Therefore, the changes observed might be achieved (although unlikely) due to the non-pharmacological life change (low-calorie diet and increased physical activity) which was recommended to the patients, and not due to GLP-1RA treatment. The absence of a control group could be, in theory, overcome by statistical correction for multiple comparisons. However, the current study was designed as a pilot and exploratory investigation aiming to evaluate the biological effect of submaximal doses of semaglutide on selected inflammatory and metabolic markers in a specific, under-researched population. As mentioned, to our knowledge, no prior study has addressed this question in non-diabetic patients with obesity receiving low-dose GLP-1 RA therapy. Given the limited sample size and hypothesis-generating intent, we decided not to apply multiple comparison correction. Although we recommended maintaining a hypocaloric diet and regular physical activity, we could not control the adherence to our recommendations, as no objective control of the adherence to non-pharmacological interventions was performed (these were monitored just by patient self-reported data during regular out-patient visits). In summary, future long-term studies with larger cohorts should be conducted to add more data to this research area. For a final answer on how submaximal doses of semaglutide affect metabolic changes and inflammation, a randomized study on larger samples and with a longer follow-up period should be designed and performed. This study should randomly assess obese patients to a standard regime treatment (low-calorie diet, exercise—ideally patient-independently controlled, cognitive-behavior therapy) and placebo versus submaximally dosed semaglutide versus maximally dosed semaglutide, and follow the patients (at least 12 months of follow-up period) for weight reduction, fat and lean body mass reduction and changes in metabolic and inflammatory markers. If adequately performed, the study designed as discussed should give a final answer regarding this interesting topic.

## 4. Methods

### 4.1. Study Design and Patients

**Figure 3:**
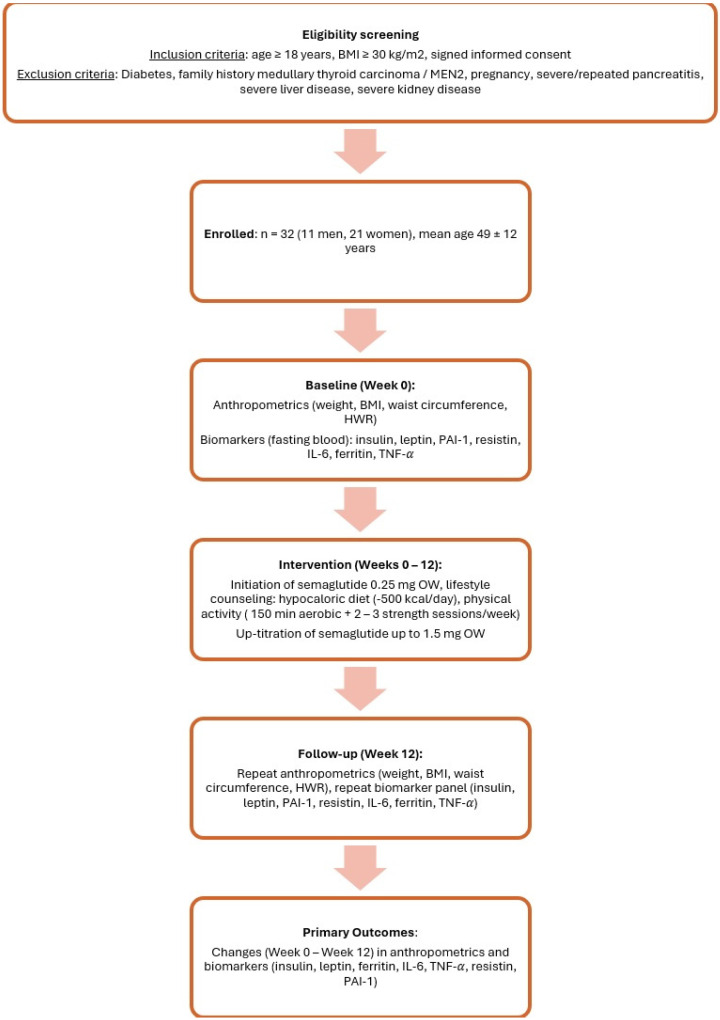
Flowchart of study design (BMI—body mass index; IL—interleukin; HWR—hip-to-waist ratio; MEN—multiple endocrine neoplasia; n—number of patients; PAI—plasminogen activator inhibitor; TNF—tumor necrosis factor).

We conducted a pilot, prospective and observational study. Our study investigated the effect of submaximal doses of long-acting GLP-1RA semaglutide on selected plasma inflammatory parameters and adipokines, namely the following: ferritin, leptin, resistin, IL-6, insulin, TNF-α and PAI-1. Consecutive presentations of patients with obesity who were treated at the obesity out-patient clinic of the Department of Internal Medicine of a Tertiary Care Hospital between 1 July 2024 to 30 December 2024 were included if they met the following inclusion criteria: age over 18 years, BMI more than 30 kg/m^2^ and signed informed consent with study participation. Furthermore, patients needed to not have met the following exclusion criteria: diabetes mellitus (type 1 and type 2, other specific types), family history of medullary thyroid carcinoma, family history of MEN2 syndrome, pregnancy, a history of severe/repeated pancreatitis, severe liver disease (stage B-C according to Child–Pugh score) and severe kidney disease (stage 4–5 according to Kidney disease improving quality outcomes—KDIGO classification). Patients enrolled in the study were treated with semaglutide at an initial dose of 0.25 mg s.c. weekly, with up-titration to a maximal dose of 1.5 mg weekly. Treatment with semaglutide was not up-titrated if the desired weight loss was achieved (2–5 kg per month). No other anti-obesity drugs were administrated. All enrolled patients were educated, and a low-calorie diet was recommended (500 kcal/day deficit). Moreover, increased physical activity (150 min of aerobic activity per week and 2–3 strength training sessions per week) was recommended. The monitoring of the adherence to calorie restriction and physical activity was checked on regular out-patient visits (once a week during the study follow-up) at our Obesity Clinic, and was based on patient self-reported data (diet diaries performed by the patients were checked by the attending physician and an interview regarding physical activity was conducted). No objective (patient-independent) monitoring of the efficacy of this intervention was performed. The research was performed according to all ethical standards, the study was approved by the local ethics committee, and, as mentioned, patients provided written informed consent prior to enrollment in the study. Blood samples as reported below were taken at baseline and after 12 weeks of submaximal semaglutide therapy. The study designed is graphically reported in Figure 3.

### 4.2. Blood Sampling and Blood Sample Analysis

After obtaining written informed consent (Figure 1), fasting venous blood samples were collected using standard Vacoutainer blood tubes with EDTA (ethylenediaminetetraacetic acid). Consequently, the specimens were centrifugated at 2500 rpm for 15 min at 4 °C (Hettich Universal 320R, Hettich, Tuttlingen, Germany). Then, the resulting plasma fractions were aliquoted and stored at −80 °C until further analysis. The Metabolic Syndrome Array 1 panel including eight biomarkers—c-peptide, ferritin, interleukin-6 (IL-6), resistin, insulin, tumor necrosis factor α (TNF-α), interleukin-1α (IL-1α), leptin and plasminogen activator inhibitor-1 (PAI-1)—was subsequently measured and quantified using biochip array technology (Evidence Investigator, Randox, Crumlin, UK), in accordance with the manufacturer’s protocol.

### 4.3. Statistical Analysis

The program Jamovi version 2.6.26.0 (Sydney, Australia) was used for data visualization and statistical analysis. Gaussian/non-Gaussian distribution of the data was evaluated using the Shapiro–Wilk test for normality. All studied parameters exhibited a nonparametric distribution and all anthropometric data exhibited a parametric distribution. A paired *t*-test (Wilcoxon rank or Student’s *t*) was used to compare the effect of therapy (adjust as necessary). The results are presented as median and interquartile range in the case of nonparametric distribution and as mean and standard deviation in the case of parametric distribution. Given the small sample size, the observational design and the aim to explore biomarker trends in response to submaximal GLP-1 RA therapy, this study was conducted as a pilot and exploratory investigation. Therefore, no correction for multiple comparisons was applied. Effect sizes and 95% confidence intervals are reported for key outcomes. *p*-values of ≤0.05 were considered statistically significant.

## 5. Conclusions

The 12-week treatment with submaximal doses of GLP-1 RA resulted in a significant reduction in body weight, waist circumference, insulin and leptin levels. Our study supports their use as part of a comprehensive obesity treatment strategy. Although we observed these significant findings, other inflammatory markers (ferritin, resistin, IL-6, TNF-α and PAI-1) did not exhibit significant reduction. In summary, GLP-1 RA has metabolic effects even at submaximal doses, particularly on insulin resistance and the adipokine profile. Future studies with larger cohorts and longer follow-ups are needed to clarify the associations.

## Figures and Tables

**Table 1:** Baseline demographic and laboratory characteristics of study cohort.

	Study Group
Age (years)	49 ± 12
Number of patients (men/women)	11/21
Dyslipidemia	27 (84.4%)
Chronic kidney disease (KDIGO stage 1–3)	3 (9.4%)
Coronary artery disease	7 (21.9%)
Atrial fibrillation	3 (9.4%)
Hypertension	17 (53.1%)
Bronchial asthma	4 (12.5%)
Valve disease—moderate to severe	3 (9.4%)
Serum creatinine (µmol/L)	71.9 ± 12.3
GFR calculated using Cockroft–Gault equation (mL/min/1.73 m^2^)	94.6 ± 14.2
ALT (µkat/L)	0.6 ± 0.2
AST (µkat/L)	0.5 ± 0.1
Hemoglobin (g/L)	144.8 ± 12.0
Total serum protein (g/L)	73.9 ± 2.3
Total cholesterol (mmol/L)	5.6 ± 1.1
LDL cholesterol (mmol/L)	3.5 ± 1.1
HDL cholesterol (mmol/L)	1.2 ± 0.3
Triglycerides (mmol/L)	2.0 ± 1.0

ALT—alanine-aminotransferase; AST—aspartate-aminotransferase; GFR—glomerular filtration rate; HDL—high-density lipoproteins; KDIGO—Kidney Disease Improving Global Outcomes; LDL—low-density lipoproteins.

**Table 2:** Selected biomarkers and anthropometric parameters before and after successful GLP-1RA treatment.

	Before Treatment	After Treatment	∆	∆ CI 95%	Significance (*p* Value)	Effect Size	CI 95%
Ferritin (ng/mL)	103 (39, 173)	107 (45.7, 156)	0.18 (−17.0, 12.2)	(−13.6, 16.9)	0.806	−0.038	(−97.96, 64.89)
IL−6 (pg/mL)	1.56 (0.8, 2.18)	1.32 (0.84, 2.18)	−0.06 (−0.46, 0.70)	(−0.33, 0.58)	0.607	−0.102	(−2.33, 2.28)
Resistin (ng/mL)	3.22 (2.68, 4.45)	3.66 (2.73, 4.88)	−0.28 (−1.06, 0.57)	(−0.66, 0.17)	0.250	0.214	(−1.85, 2.04)
Insulin (uIU/mL)	21.6 (15.1, 42.3)	13 (9.39, 22.3)	6.46 (2.49, 19.10)	(6.43, 26.5)	<0.001	−0.592	(−77.46, 15.49)
TNF-alpha (pg/mL)	4.03 (3.16, 4.8)	4.1 (3.17, 5.06)	−0.07 (−0.61, 0.43)	(−0.59, 0.39)	0.633	0.074	(−2.11, 2.65)
Leptin (ng/mL)	24.6 (15.3, 35.7)	14.1 (7.71, 27.1)	7.80 (1.82, 16.70)	(4.39, 14.40)	<0.001	−0.675	(−39.74, 17.15)
PAI-1 (ng/mL)	4.92 (2.23, 14.6)	5.07 (3, 8.89)	1.75 (−2.25, 6.07)	(−1.70, 4.38)	0.134	−0.159	(−15.79, 15.76)
Weight (kg)	118 ± 24.6	106 ± 24.3	12 ± 6.3	(9.1, 13.5)	<0.001	1.78	(1.23, 2.32)
BMI (kg/m^2^)	40.5 ± 7.3	36.7 ± 7.41	3.8 ± 2.41	(2.9, 4.6)	<0.001	1.56	(1.05, 2.05)
Waist circumference (cm)	121 ± 15.6	114 ± 15.6	8 ± 7.2	(5.0, 10.0)	<0.001	1.05	(0.62, 1.46)
Height/waist ratio	0.71 ± 0.09	0.67 ± 0.09	0.04 ± 0.04	(0.03, 0.06)	<0.001	1.03	(0.61, 1.44)

BMI—body mass index; GLP-1RA—glucagon-like peptide-1 receptor agonists; PAI-1—plasminogen activator inhibitor-1; TNF—tumor necrosis factor.

## Data Availability

All data are available from the corresponding author upon a reasonable request. The data are not publicly available due to legal restrictions.
